# Miscellaneous Items

**Published:** 1886-06

**Authors:** 


### MISCELLANEOUS ITEMS.

**One pound** of cheese has more nutriment in it than three pounds of beef.

**The early Greeks** were vegeterians. They did not degenerate until most of them became gluttons.

**Inhalations** of steam impregnated with oil of peppermint afford great relief in painful throat affections.

**A Hospital** for the reception and care of indigent patients was dedicated with due ceremony, at Reading, Pa., on the 10th inst.

**It is reported** that Dr. Keith was recently called from Edinburgh to Boston in consultation, and that he received for it a fee of $10,000.

**A diet** of grains, good brown bread, milk and fruits, will give your children sweet breath, white teeth, healthy digestive organs and general good health.

**Empirical** medicine, thank heaven, has had its day, and the sooner it becomes a theory of the past the better it will be for the whole world.—*C. B. Steman, M.D.*

**The American** Institute of Homœopathy, the oldest National medical organization in the United States, meets at Saratoga, N. Y. June 28th, and remains in session four days.

**A number** of cures of cases of sciatica and local neuralgia are reported from Paris. The means employed was the application of a freezing mixture to the skin directly over the pain.

**People** who have been carelessly eating pineapples, believing them to be harmless, will be pleased to learn that the rind of the fruit contains an acrid poison which is likely to cause sore mouth and lips. In Cuba salt is used as an antidote.

**Dr. E. D. Babbitt,** of 20 University Place, this city, is the author of the article on Wonders of the Sunlight in this number, as well as of various works on Light and Color. He is the founder of the sun-healing movement, and has devised instruments in which solar-sweat-baths can be given in a scientific manner.

**The vocation** of the highwayman, who obtains his victim's money at the mouth of a pistol, is an honorable one as compared with that of the charlatan who obtains his from the almost exhausted purse of the invalid, by taking advantage of his ignorance and of his desire for health.—*Fort Wayne Journal of Medical Science.*

**Asphaltum Varnish.**—Dr. Borck, of St. Louis, says that asphaltum varnish is the best disinfectant he knows of ; it will destroy all germs at once, and no household insects will approach an article of furniture whose interior has been painted with it.

**The Phrenological Journal** for June, has an unusual amount of excellent reading for all classes. It is so full of good things for the people that we cannot find room for a notice for them all. The Editorial and Correspondence departments are replete with mental food of good quality. To the student of human nature, the perplexed parent, the earnest teacher, and the aggressive preacher this number of the *Journal*, has a particular value ; in fact every number contains invaluable hints and instruction for every day use. While aggressive in its teachings, it is noticeable that there is no bigotry displayed, no "I am right, and all the rest of the world is wrong," in its columns. The arguments in favor of Phrenology, Hygiene, etc., are not of the "Shillalah," class but built after the model recommended by King Solomon. The subscription price is $2.00 per year, or 20 cts. per number.

FOWLER & WELLS Co., Publishers,
753 Broadway, New York.

**Vicks Illustrated Monthly** comes promptly to hand, and is filled with valuable information and entertainment for all who love the flowers and the garden.

**Dr. Livezy** writes, "While wintering in Florida I met with my annual patient, a young lady of twenty-eight, from Chicago, who was sent hither three or four years ago in order to pass into the "spirit land" comfortably, who was then troubled with poor appetite, a slight but distressing nausea, great debility, irregular menstruation, excessive cardiac action on the least exertion, etc. I ordered a 1-oz. bottle of Lactopeptine of the N. Y. Pharmacal Association's manufacture and she improved at once. Soon after she met a lady friend, who told her she ought to take Lactopeptine, stating what wonders it had done for her, who was troubled "just the same way" (of course). "Why, bless me," said my patient, "that is just what my doctor prescribed for me and I am doing nicely." By the time she finished the small vial she declared she never felt better in her life, her appetite being regular and everything O. K. N. B.—She has taken since Lactopeptine, Elixir, Calisaya, Iron and Bismuth, with excellent results. —*Medical Summary.*

